# Metabolomics Reveals New Mechanisms for Pathogenesis in Barth Syndrome and Introduces Novel Roles for Cardiolipin in Cellular Function

**DOI:** 10.1371/journal.pone.0151802

**Published:** 2016-03-25

**Authors:** Yana Sandlers, Kelly Mercier, Wimal Pathmasiri, Jim Carlson, Susan McRitchie, Susan Sumner, Hilary J. Vernon

**Affiliations:** 1 Department of Chemistry, Cleveland State University, Cleveland, OH, United States of America; 2 Research Triangle International, Durham, NC, United States of America; 3 Department of Neurogenetics, Kennedy Krieger Institute, Baltimore, MD, United States of America; 4 McKusick-Nathans Institute of Genetic Medicine, Johns Hopkins University, Baltimore, MD, United States of America; National Research Council of Italy, ITALY

## Abstract

Barth Syndrome is the only known Mendelian disorder of cardiolipin remodeling, with characteristic clinical features of cardiomyopathy, skeletal myopathy, and neutropenia. While the primary biochemical defects of reduced mature cardiolipin and increased monolysocardiolipin are well-described, much of the downstream biochemical dysregulation has not been uncovered, and biomarkers are limited. In order to further expand upon the knowledge of the biochemical abnormalities in Barth Syndrome, we analyzed metabolite profiles in plasma from a cohort of individuals with Barth Syndrome compared to age-matched controls via ^1^H nuclear magnetic resonance spectroscopy and liquid chromatography-mass spectrometry. A clear distinction between metabolite profiles of individuals with Barth Syndrome and controls was observed, and was defined by an array of metabolite classes including amino acids and lipids. Pathway analysis of these discriminating metabolites revealed involvement of mitochondrial and extra-mitochondrial biochemical pathways including: insulin regulation of fatty acid metabolism, lipid metabolism, biogenic amine metabolism, amino acid metabolism, endothelial nitric oxide synthase signaling, and tRNA biosynthesis. Taken together, this data indicates broad metabolic dysregulation in Barth Syndrome with wide cellular effects.

## Introduction

Barth Syndrome (BTHS, 3-methylglutaconic aciduria type II, MIM 300394) is a rare X-linked disorder caused by defects in *TAZ* (G4.5), which encodes for Tafazzin, an acyltransferase involved in the remodeling of the mitochondrial phospholipid cardiolipin [1,2]. Deficiency of tafazzin results in abnormal cardiolipin content and a reduction of mature cardiolipin.

Cardiolipin is one of the predominant phospholipids of the mitochondrial inner membrane and tetralinoleyl-cardiolipin is the most prevalent form in human heart and skeletal muscle mitochondria [3,4]. Cardiolipin has important roles in mitochondrial function including maintaining christae structure, supporting electron transport chain efficiency, and in apoptosis [4–7]. Abnormalities in cardiolipin have been implicated in common human diseases including diabetes, heart failure, and Parkinson’s disease, and with high-lipid dietary intake [8–10]. However, Barth Syndrome (BTHS) is the only known Mendelian disorder of cardiolipin remodeling [1,2]. The characteristic clinical features of BTHS include cardiomyopathy, skeletal myopathy, and intermittent neutropenia, though some clinical variability does exist [11]. Females are not known to be affected on a clinical or biochemical level [12].

The primary diagnostic metabolite measurement in Barth Syndrome is elevation of the monolysocardiolipin to cardiolipin ratio (MLCL/CL ratio). This ratio has a high diagnostic sensitivity and specificity measured in bloodspots, nucleated cells, and tissues [13]. Elevations of 3-methylglutaconic aciduria are also often found in blood and urine of individuals with BTHS, though normal values have been reported even in severely affected individuals [14–17].

While the primary biochemical cardiolipin defect in BTHS has been defined, many downstream metabolic abnormalities remain unsolved, and thus targets for treatment and clinical monitoring are limited. Previous metabolomics studies by our group evaluated plasma amino acids, plasma 3-methylglutaconic acid, intermediates of cholesterol synthesis, and red blood cell membrane fatty acid profiles in a cohort of individuals with Barth Syndrome. A unique biochemical profile that differentiates subjects with BTHS from age-matched controls was uncovered, including decreased plasma arginine, increased proline, decreased omega-6 fatty acids, and increased saturated fatty acids [12].

In the present study, we employed two complementary metabolomics techniques, nuclear magnetic resonance spectroscopy and liquid chromatography mass spectrometry, in order to characterize the differences in metabolic profiles of plasma from individuals with BTHS and age-matched controls. We uncovered discriminating metabolites involved in multiple mitochondrial and extra-mitochondrial biochemical pathways including broad effects on cellular lipid metabolites. These results lay the groundwork for exploration of novel cellular mechanisms in BTHS, and introduce potential new biomarkers for disease monitoring.

## Materials and Methods

### Study Subjects

This study was carried out using plasma from 23 individuals with biochemically or molecularly confirmed diagnosis of Barth Syndrome and 15 age-matched control samples from individuals not known to have an inborn error of metabolism. The study was approved under Johns Hopkins University IRB protocols “NA_00090474, Multidisciplinary studies in Barth Syndrome” and “NA_00069372, Metabolic analysis of archived biofluid samples”. For the individuals with Barth Syndrome, written consent was obtained from the donor or the next of kin for the use of samples in this research under the “Multidisciplinary studies in Barth Syndrome” protocol. For the control samples, our institutional review board waived the need for consent under the “Metabolic analysis of archived biofluid samples” protocol.

Samples were collected 3–4 hours post-prandial to account for potential metabolic effects of a recent meal.

### NMR Metabolomics

Sample preparation, data acquisition, statistics, and pathway analysis were performed as previously described [18–24]. Each plasma sample (125 μL) was prepared by addition of a 0.9% saline solution containing 2 mM formate (chemical shift indicator). In addition, a plasma pooled sample was prepared by mixing 12 μL of each study sample. Three 125 μL aliquots of the pooled plasma were prepared identical to the individual plasma samples. Metabolomics data were acquired for each of the individual study samples and the pooled samples. ^1^H NMR spectra of plasma samples were acquired on a Bruker Avance III 950 MHz NMR spectrometer (located at the David H. Murdock Research Institute at Kannapolis, NC, USA) using a CPMG pulse sequence. NMR spectra were pre-processed using ACD 1D NMR Processor 12.0 (ACD Labs, Toronto, Canada). NMR bins (0.75–7.75 ppm) were made after excluding water (4.15–5.15 ppm) using intelligent binning width of 0.04 ppm and 50% looseness factor. Integrals of each of the bins were normalized to total integral of each of the spectrum.

### LC-MS Targeted Metabolomics

Targeted metabolomics was conducted using electrospray ionization liquid chromatography–mass spectrometry (ESI-LC-MS/MS) and MS/MS measurements using the Absolute*IDQ*™ p180 kit (Biocrates Life Sciences AG, Innsbruck, Austria). This kit simultaneously quantifies 188 metabolites in 10 μL of plasma, including free carnitine, 40 acylcarnitines (Cx:y), 21 amino acids (19 proteinogenic amino acids, citrulline and ornithine), 21 biogenic amines, hexose (sum of hexoses–about 90–95% glucose), 90 glycerophospholipids (14 lysophosphatidylcholines (lysoPC) and 76 phosphatidylcholines (PC diacyl (aa) and acyl-alkyl (ae)), and 15 sphingolipids (SMx:y). The assay procedures of the Absolute*IDQ*™ p180 kit as well as the metabolite nomenclature have been described in detail previously [25–26].

Mass spectrometric (MS) analyses were carried out on an API 4000 LC-MS/MS System (AB Sciex, Framingham, MA) equipped with 1100 Series HPLC (Agilent Technologies, Palo Alto, CA) using an Agilent Eclipse XDB-C_18_ (3.5 μm) 3.0 x 100 mm column controlled by Analyst 1.6.2 software. Multiple Reaction Monitoring (MRM) was used for the detection of analytes and stable labeled internal standards, the latter were used as quantification reference. The acquired data was processed using Analyst 1.6.2 and Met*IDQ* (Biocrates Life Sciences AG, Innsbruck, Austria) software. Concentrations of all metabolites were calculated in μM.

### Multivariate and Statistical Analysis

Descriptive statistics and two-sided t-tests, using the Satterthwaite approximation for unequal variances, were conducted for the binned NMR data and targeted LC-MS data using SAS 9.4 (SAS Institute Inc, Cary, NC).

Targeted LCMS concentration data was used to conduct multivariate analysis with UV scaling for each of the measured metabolites and custom ratios. Normalized binned NMR data were mean centered and Pareto scaled prior to multivariate analysis. Multivariate data analysis methods (e.g. principal component analysis [PCA], orthogonal partial least squares discriminant analysis [OPLS-DA]) were used to reduce the dimensionality and to enable the visualization of the separation of the study groups (SIMCA 13, Umetrics, Umeå, Sweden). The PCA plots were inspected to ensure that the pooled samples were tightly clustered in the center of all of the individual study samples, a quality control method that is widely used in metabolites studies [27]. Samples observed to fall outside the 99% confidence interval were evaluated for possible removal from the multivariate analysis. In addition, the distance to the model in the X-data (DModX) was compared to the critical distance for the 95% confidence interval based on the F distribution, and samples with a distance greater than twice the critical distance were also evaluated for possible removal [28, 29]. All models used a 7-fold cross-validation to assess the predictive ability of the model (Q^2^). Loadings plots and variable influence on projections (VIP) plots were inspected, and bins that had a VIP ≥ 1.0 with a jack-knife confidence interval that did not include 0 were determined to be important to differentiating the study groups. Chenomx NMR Suite 7.7 Professional software (Edmonton, Alberta, Canada), which has a concentration library of approximately 350 compounds, was used to match the signals in the identified bins to metabolites.

### Pathway Analysis

Library matched metabolites identified by NMR as important to distinguishing subjects (VIP ≥ 1.0) with BTHS from controls and metabolites identified by targeted LC-MS analysis as significantly different between BTHS and controls (p < 0.05) were analyzed for pathway enrichment analysis using the knowledge-based canonical pathways and endogenous metabolic pathways in the MetaCore module in GeneGo software (Chicago, IL). Ranking of relevant pathways was based on hypergeometric p-values. In addition, networks were built using the metabolites identified by NMR and LC-MS as important to differentiating the study groups and Tafazzin as network objects with the trace pathways algorithm (with 3 maximum steps in pathways).

## Results

Subjects with BTHS (n = 23), had a mean age of 147.4 months ± 115 (SD) (range 6 months-32 years) and controls (n = 15) had a mean age of 117.1 months ±99 (SD) (range 6 months-26 years). Body mass index (BMI) was available for 19/23 individuals with BTHS, with a mean of 17.1 kg/m^2^ ±4.0 (SD) and for 13 controls with a mean of 18.7 kg/m^2^ ±4.4 (SD). All samples were from male participants. There was no statistical significance in BMI or age between cases and controls.

Four of the control samples were removed from the ^1^H-NMR spectroscopy analysis due to potential EDTA contamination, and one of the BTHS samples was removed from the ^1^H-NMR spectroscopy analysis due to poor water suppression. Removal of these samples from the control set did not cause a statistically significant difference in either age or BMI averages. All samples were included in the Biocrates analysis, as the targeted multiple-reaction-monitoring (MRM) LCMS approach will selectively monitor metabolite signals in the presence of background compounds.

### Broad Spectrum NMR Metabolomics

**Fig 1:**
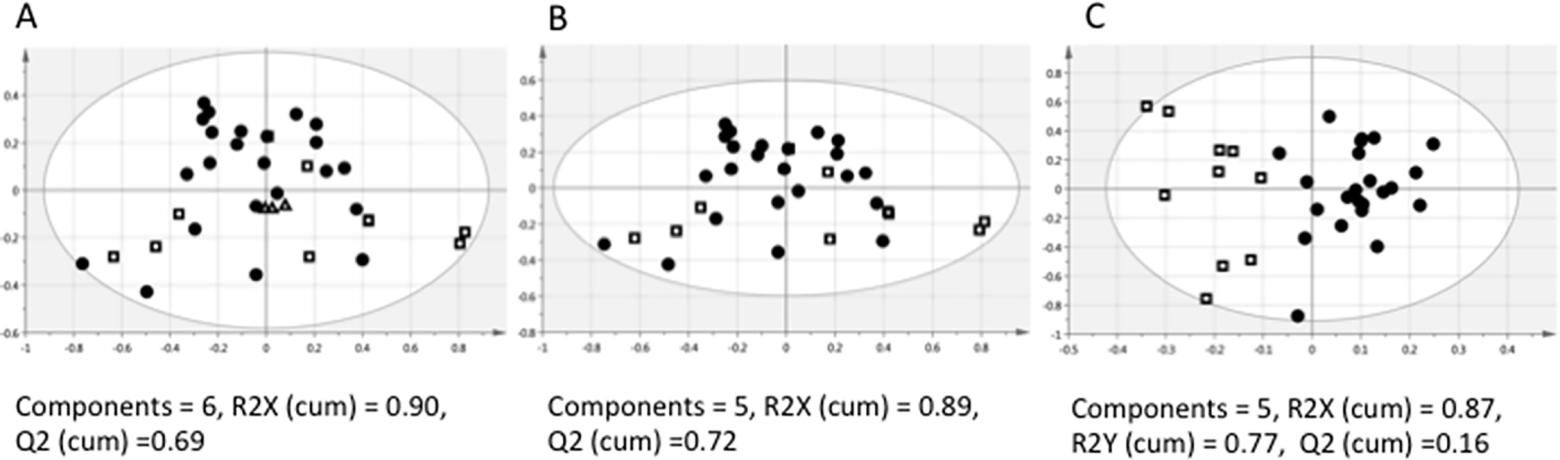
Multivariate scores plots for the binned ^1^H NMR data (BTHS are filled circles, Controls are empty squares, and total pools are empty triangles). (A) PCA plot for the 33 study samples and the three pooled samples created as quality control samples. The total pools are tightly clustered and centered indicating high quality sample preparation and data acquisition. (B) PCA plot of the 33 study samples. (C) BTHS and controls differentiated with 100% accuracy in the supervised (OPLS-DA) model (Fisher’s probability 1.1 x 10^−8^, n = 33, 1 predictive component and 4 orthogonal components, R^2^X (cum) = 0.87, R^2^Y (cum) = 0.77, Q^2^ (cum) = 0.16).

Fig 1A shows a PCA plot of the binned NMR data for the 33 study samples and the three pooled samples created as quality control samples. The total pools are tightly clustered and centered, indicating high quality sample preparation and data acquisition. The PCA plot of the binned data for the 33 study samples (Fig 1B) shows all samples lie within the ellipse based on Hotelling’s T^2^ 95% confidence interval. There is some clustering, but BTHS and control samples are not well differentiated in the unsupervised analysis (n = 33, 5 components, R^2^X (cum) = 0.89, Q^2^ (cum) = 0.72). The OPLS-DA plot (Fig 1C) shows that one control sample is just outside the edge of the ellipse for the 95% confidence interval. The maximum distance to the model in the X data (DModX) was 1.8, which is less than twice the critical distance (2*1.27 = 2.54) defined by the F distribution. Therefore, no samples were identified as moderate or strong outliers, and all 33 study samples were included in the multivariate analyses for the NMR binned data. BTHS and controls differentiated with 100% accuracy (Fisher’s probability 1.1 x 10^−8^, n = 33,1 predictive component and 4 orthogonal components, R^2^X (cum) = 0.87, R^2^Y (cum) = 0.77, Q^2^ (cum) = 0.16).

Library matched metabolites that were identified as being important (VIP ≥ 1.0 with a jack-knife confidence interval that did not include 0) to distinguishing Barth Syndrome samples from controls included: 3-hydroxybutyrate, creatinine, carnitine, lipids/fatty acids, very low density lipoproteins (VLDLs), methionine, proline, unsaturated lipids/fatty acids (S1 Table). Pathway enrichment analysis using the library-matched metabolites that were identified by NMR as being important differentiators include signaling in cholinergic neurons, insulin regulation of fatty acid metabolism, and pancreatic beta cell differentiation and function (Table 1).

**Table 1:** Top ten statistically significant pathway maps created using GeneGo enrichment analysis based on library-matched metabolites that differentiate (VIP ≥ 1) patients with BTHS from controls. (FDR = false discovery rate).

Rank	Maps	p-value	FDR
1	Nicotine signaling in cholinergic neurons	8.018E-06	4.891E-04
2	Regulation of lipid metabolism/Insulin regulation of fatty acid metabolism	4.573E-05	1.395E-03
3	Role of prenatal nicotine exposure in inhibition of pancreatic beta cells differentiation and function	1.347E-04	2.146E-03
4	Amitraz-induced inhibition of Insulin secretion	1.481E-04	2.146E-03
5	N-Acylethanolamines, HRASLS-transacylation pathway	2.245E-04	2.146E-03
6	Possible influence of low doses of Arsenite on glucose uptake in muscle	2.417E-04	2.146E-03
7	Influence of low doses of Arsenite on glucose uptake in adipocytes	2.969E-04	2.146E-03
8	Acetylcholine biosynthesis and metabolism	3.166E-04	2.146E-03
9	Phospholipid metabolism p.3	3.166E-04	2.146E-03
10	Immune response_IL-7 signaling in T lymphocytes	4.476E-04	2.730E-03

### Targeted Metabolomics

Based on previous metabolomics results, which implicated fatty acid and amino acid perturbations, quantitative measurements were obtained using the Biocrates assay, which simultaneously quantifies 188 metabolites, including lipids and specific metabolites representative of pathways of interest. A supervised multivariate analysis (OPLS-DA) of all metabolites detected via this assay showed a clear distinction between cases and controls (S1A Fig). When evaluated as individual metabolite groups, multivariate analysis of acylcarnitines, amino acids, biogenic amines, and glycerophospholipids also showed a clear distinction between BTHS and controls (S1B, S1C, S1D and S1E Fig). Sphingolipids showed a distinction as well, but BTHS and controls were less well separated than the other metabolite groups (S2 Table). Outliers were expected in the multivariate analyses of the targeted data due to sample diversity, and only the most extreme outlier identified in the biogenic amines analysis was removed from the final OPLS-DA model.

The amino acids that were most significantly different (p < 0.05) between cases and controls were proline (1.5 fold increase in cases), arginine (1.2 fold decrease in cases) and tyrosine (1.4 fold increase in cases). This is strikingly similar to the pattern of amino acid changes seen in our previous study, and further validates these as significant findings [11]. This assay revealed a wide array of other compounds and custom ratios that were either statistically significant (p < 0.05) between BTHS and controls or where the magnitude of the fold change was greater than 2 (S3 Table). One statistically significant metabolite (p < 0.05) important in differentiating BTHS and controls in the multivariate analysis was serotonin (S3 Table), which was found to be 8.2 fold lower in BTHS than controls.

### Combined modality data analysis

Marker metabolites identified in the multivariate data analysis of the NMR metabolomics data and those identified in the Biocrates analysis were used in the pathway enrichment analysis which was performed using the knowledge-based canonical pathways and endogenous metabolic pathways in the MetaCore module of the GeneGo software. Ranking of relevant pathways was based on hypergeometric *p*-values and include: aminoacyl-tRNA biosynthesis in mitochondria and cytoplasm, regulation of endothelial nitric oxide synthase (eNOS) activity in endothelial cells, regulation of lipid metabolism, and transport of intracellular cholesterol (Table 2).

**Table 2:** Top ten statistically significant pathway maps created using GeneGo enrichment analysis from the NMR and MS p180 Biocrates analyses that are important to differentiating patients with BTHS from controls (VIP ≥ 1). (FDR = false discovery rate).

Rank	Maps	p-value	FDR
1	Aminoacyl-tRNA biosynthesis in mitochondrion	9.22E-08	1.22E-05
2	Aminoacyl-tRNA biosynthesis in cytoplasm	2.72E-07	1.27E-05
3	Aminoacyl-tRNA biosynthesis in cytoplasm/ Rodent version	2.89E-07	1.27E-05
4	Nociception/Pro-nociceptive action of Nociceptin in spinal cord at low doses	5.83E-06	1.92E-04
5	Muscle contraction/Regulation of eNOS activity in endothelial cells	3.37E-05	8.90E-04
6	Regulation of lipid metabolism/PPAR regulation of lipid metabolism	2.32E-04	5.10E-03
7	Transport_Intracellular cholesterol transport	1.71E-03	3.15E-02
8	Apoptosis and survival_NO signaling in apoptosis	1.98E-03	3.15E-02
9	Apoptosis and survival_NO signaling in survival	2.15E-03	3.15E-02
10	Glycine links	2.90E-03	3.45E-02

In order to determine whether plasma metabolites can directly connect to factors that contribute to Barth Syndrome, the trace pathways network option in MetaCore with no more than three steps was used. *TAZ*, the gene underlying Barth Syndrome, was also included in the network. The analysis revealed that many metabolites identified by NMR and the Biocrates analysis were incorporated in the same network as the *TAZ*. These metabolites identified in the 3-step network analysis were 2-aminoadipic acid, 3-hydroxybutyric acid, arginine, carnitines, carnosine, citrulline, creatinine, fatty acids, glycine, LysoPCs, methionine, PCs, proline, serotonin, taurine, SMs, VLDL lipids, tyrosine, and valine (S2 Fig).

## Discussion

**Fig 2:**
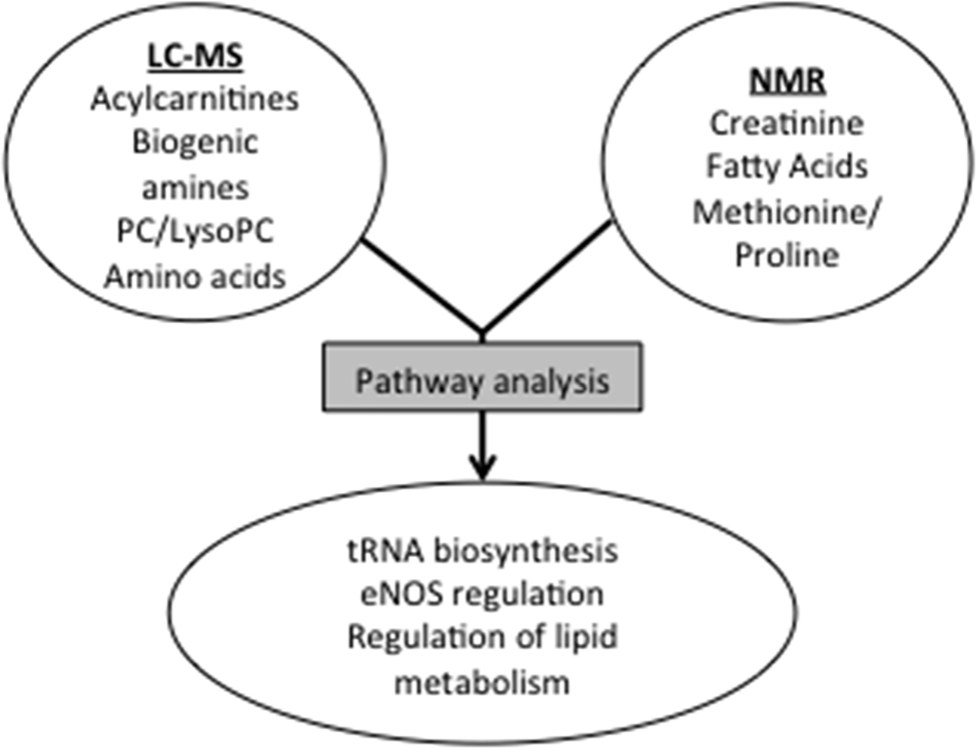
Metabolic pathway analysis of combined NMR and LC-MS data.

In the present study, we have identified plasma metabolites that are important in distinguishing individuals with BTHS from controls through untargeted and targeted metabolomics analysis, including: amino acids, lipids, biogenic amines, and acylcarnitines. Pathway analysis of these distinguishing metabolites suggests a wide range of mitochondrial and extra-mitochondrial cellular dysregulation, including regulation of lipid metabolism, aminoacyl-tRNA biosynthesis, regulation of nitric oxide synthase, and other pathways (Fig 2).

We previously described significant differences in plasma amino acids in a cohort of individuals with BTHS compared with controls including increased proline, tyrosine, and asparagine and decreased arginine and cysteine [12]. Reduced arginine was also reported in a separate cohort of BTHS patients [17]. This current study also showed a similar profile, with significantly increased amino acids including proline and tyrosine, and significantly decreased arginine in BTHS compared with controls. The increased proline levels probably reflect the chronic lactic acidemia found in many patients with BTHS. It has been postulated that arginine, as an important precursor of 2-ketoglutarate, is utilized and thus depleted as an anapleurotic metabolite in the citric acid cycle in BTHS as a result of the mitochondrial dysfunction induced by abnormal cardiolipin content[12, 17]. In addition, arginine is a major precursor for nitric oxide, which plays an important role in vascular smooth muscle function and vascular patency [30]. Arginine supplementation has been implicated as an important component of care in other mitochondrial conditions including mitochondrial encephalomyopathy, lactic acidosis, and stroke-like episodes (MELAS), and has been shown to improve outcome of neurologic events [31]. This study provides evidence for the potential importance of remediating potential arginine depletion in BTHS patients, where vascular health has particular implications in the setting of cardiac disease.

In this study, phosphatidylcholines (PC) and lysophosphatidylcholines (LysoPC) were among the metabolites that were important for distinguishing Barth Syndrome patients from control subjects. We hypothesize that these PCs and LysoPCs were differentially expressed due to abnormalities in cardiolipin metabolism. Cellular lipid metabolism involves a fluid and dynamic set of biochemical exchanges. It is therefore not surprising that abnormal cardiolipin metabolism has the potential to effect extra-mitochondrial cellular functions due to the ongoing communication between cellular compartments [32]. In line with these findings, abnormal choline diacylglycerophospholipids have been identified in *TAZ* knockdown mice, and abnormalities have been reported in total red blood cell fatty acids of Barth Syndrome patients [12, 33].

Interestingly, the overall PC and LysoPC content in BTHS patients compared to controls indicates an elevated PC/LysoPC ratio (p-value 0.029, 1.4 fold difference) (S3 Table). This ratio has been previously implicated as a marker for disease processes ranging from rheumatoid arthritis, where an increased serum PC/LysoPC ratio was shown to correspond to response to anti-inflammatory therapy, to Alzheimer’s disease, where affected individuals were found to have an increased CSF PC/LysoPC ratio, thought to be downstream effects of alterations in the metabolism of choline-containing phospholipids [34, 35]. Clarification of the etiology of this ratio alteration in BTHS could have important implications for disease and therapy monitoring.

One of the metabolites with the highest magnitude of fold change in the targeted study was a decrease in plasma serotonin in BTHS cases versus controls. Serotonin is an interesting metabolite with roles in multiple physiologic processes with potential mechanistic relevance to BTHS in the periphery including vasoconstriction, cardiomyocyte growth, and insulin secretion [36–38]. However, plasma serotonin levels are notoriously difficult to measure due to multiple confounding sample processing and assay factors [39]. With the processing methodology in our sample collection, it is impossible to rule out platelet contamination (platelets as a rich source of serotonin) as a confounder. Many labs try to circumvent the difficulties in measuring plasma serotonin by measuring the levels of the serotonin precursor 5-hydroxyindole-acetic acid (5-HIAA) in urine. We did perform this assay in a separate cohort of urine samples from other individuals with BTHS compared to controls and found no differences (data not shown). Therefore, additional studies are required to validate this finding.

Pathway analysis of the most significant metabolites detected by both broad spectrum and targeted metabolomics suggested dysregulation in both intra- and extra-mitochondrial aminoacyl tRNA biosynthesis. Interestingly, signals in this same pathway were also seen in transcriptomic analysis from BTHS mutant mouse cardiac tissue, further validating our metabolomics analysis [33]. tRNA biosynthetic abnormalities have been reported as a response to cellular stress, including oxidative and nutritional stress [40]. This stress response appears to fit with BTHS as a mitochondrial disorder.

In exploring common mechanistic underpinnings of the metabolic pathways implicated in this study, a general dysregulation of lipidation of G-protein coupled receptors (GPCRs) could potentially explain the connection between widespread lipid metabolism abnormalities and serotonin, acetylcholine, fatty acid, and insulin metabolism. GCPRs are highly post-translationally modified cell signaling hubs, with diverse cellular functions and connect to both serotonin in the extracellular region and Tafazzin (S2 Fig). They are highly sensitive to their lipid environment [41]. Different GPCR classes are affected by lipid modifications including dynamic palmitoylation, myristoylation, and isoprenylation [41]. Acetylcholine receptors, free fatty acid receptors and some serotonin receptors all involve lipidated GPCRs, and insulin regulation is secondarily affected by metabolites using lipidated GPCRs including glucagon and flotillin [42–44]. GPCRs also play a large role in eNOS signaling [45]. Interestingly, other GPCRs affect taste sensation and longitudinal growth, two other areas which are clinically abnormal in Barth Syndrome patients [46,47]. This area requires further investigation including functional GPCR analysis. However, confirmed dysfunction in these GPCR pathways would provide a framework for more intensive clinical monitoring of associated health complications (e.g. diabetes), and thus earlier treatment should these complications arise.

By investigating the disrupted metabolism in plasma of individuals diagnosed with BTHS, we have offered novel targets for further exploration in this disorder. One limitation of this study is that the metabolite profiles were obtained from plasma, which represents the extracellular space, and does not entirely reflect intracellular metabolism. Mechanistic *in vitro* studies are warranted to clarify the precise nature of these pathway abnormalities, including tRNA biosynthesis abnormalities, generalized cellular lipid dysregulation and GPCR signaling. Additionally, the findings in this study could be extended to analyses of other conditions that converge on the cardiolipin pathway and offer insight into those more common diseases.

## Supporting Information

S1 Table The NMR bins that were identified by the supervised multivariate analysis (OPLS-DA) through Chenomx library matching as being important for differentiating Barth Syndrome metabolomics profiles from those of the age-matched control profiles. Bins can contain more than one metabolite. P values are based on the t-test using the Satterthwaite approximation for unequal variances. A positive fold change indicates that the mean concentration of the analyte was higher in BTH than controls.
(DOCX)

S2 Table The metabolites identified in the targeted analysis using the P180 Biocrates Kit by the supervised multivariate analysis (OPLS-DA) as being important for the differentiating Barth Syndrome metabolomics profiles from those of the age-matched control profiles. (DOCX)

S3 Table The metabolites and custom ratios quantified by LC-MS using the p180 Biocrates Kit that were either statistically significant (p < 0.05) between BTHS and controls based on the t-test using the Satterthwaite approximation for unequal variances or where the magnitude of the fold change was greater than 2. A positive fold change indicates that the mean concentration of the analyte was higher in BTHS than controls.
(DOCX)

S1 Fig Supervised analysis (OPLS-DA) of LC-MS data showing a clear separation between cases (circles) and controls (squares). (A) All LC MS/MS data combined, (B) acylcarnitines, (C) amino acids, (D) biogenic amines, and (e) glycerophospholipids. One sample was identified as an extreme outlier and excluded from the biogenic amines analysis
(TIFF)

S2 Fig GeneGo 3-step Network analysis connects Tafazzin to G-protein coupled receptors and extracellular serotonin. The purple circled metabolites are those that were identified as VIPs in the metabolomics analysis. The center of the map highlights the connectivity between the Tafazzin, GPCRs, fatty acids, and serotonin.
(TIFF)
