# Supplementary material for: Metabolomics Reveals New Mechanisms for Pathogenesis in Barth Syndrome and Introduces Novel Roles for Cardiolipin in Cellular Function
Source: PLoS One. 2016 Mar 25;11(3):e0151802. doi: 10.1371/journal.pone.0151802 (PMC4807847; doi:10.1371/journal.pone.0151802)
Supplement: S1 Table — Bins can contain more than one metabolite. P values are based on the t-test using the Satterthwaite approximation for unequal variances. A positive fold change indicates that the mean concentration of the analyte was higher in BTH than controls. (DOCX) [file pone.0151802.s001.docx]

Supplementary Table 1. The NMR bins that were identified by the supervised multivariate analysis (OPLS-DA) through Chenomx library matching as being important for differentiating Barth Syndrome metabolomics profiles from those of the age-matched control profiles. Bins can contain more than one metabolite. P values are based on the t-test using the Satterthwaite approximation for unequal variances. A positive fold change indicates that the mean concentration of the analyte was higher in BTH than controls.

| Library-Matched Metabolite Bins | VIP | p-value | Fold Change |
| --- | --- | --- | --- |
| 3-Hydroxybutyrate | 2.2 | 0.005 | 1.8 |
| Carnitine, Lipids/Fatty Acids | 2.8 | 0.026 | 1.2 |
| Creatinine, Overlap | 1.5 | 0.001 | -1.4 |
| Lipids/Fatty Acids (4 bins) | 1 - 2.3 | 0.012 - 0.062 | (-1.6) - 1.2 |
| Methionine | 1.8 | 0.003 | 1.2 |
| Proline, Lipids/Fatty Acids | 1.4 | 0.142 | -1.1 |
| Unsaturated Lipids/Fatty Acids (2 bins) | 1.5 - 1.9 | 0.078 - 0.154 | (-1.5) - (-1.3) |
| VLDLs | 4.3 | 0.023 | -1.4 |
| VLDLs, Unknown | 5.1 | 0.136 | -1.3 |
