# Supplementary material for: Metabolomics Reveals New Mechanisms for Pathogenesis in Barth Syndrome and Introduces Novel Roles for Cardiolipin in Cellular Function
Source: PLoS One. 2016 Mar 25;11(3):e0151802. doi: 10.1371/journal.pone.0151802 (PMC4807847; doi:10.1371/journal.pone.0151802)
Supplement: S2 Table — (DOCX) [file pone.0151802.s002.docx]

Supplementary Table 2. The metabolites identified in the targeted analysis using the P180 Biocrates Kit by the supervised multivariate analysis (OPLS-DA) as being important for the differentiating Barth Syndrome metabolomics profiles from those of the age-matched control profiles.

| Classification | Analyte* | VIP |
| --- | --- | --- |
| Acylcarnitines | C2 | 1.6 |
| Acylcarnitines | C3-DC (C4-OH) | 1.6 |
| Acylcarnitines | C14 | 1.5 |
| Acylcarnitines | C4 | 1.4 |
| Acylcarnitines | C12:1 | 1.2 |
| Acylcarnitines | C5:1 | 1.2 |
| Acylcarnitines | C16 | 1.2 |
| Acylcarnitines | C14:1 | 1.1 |
| Acylcarnitines | C6 (C4:1-DC) | 1.1 |
| Acylcarnitines | C9 | 1.1 |
| Acylcarnitines | C14:2 | 1.1 |
| Acylcarnitines | C5-DC (C6-OH) | 1.0 |
| Acylcarnitines | C10:1 | 1.0 |
| Acylcarnitines | C16:1 | 1.0 |
| Acylcarnitines | C12 | 1.0 |
| Amino Acids | Proline | 1.5 |
| Amino Acids | Arginine | 1.4 |
| Amino Acids | Tyrosine | 1.3 |
| Amino Acids | Asparagine | 1.3 |
| Amino Acids | Methionine | 1.2 |
| Amino Acids | Phenylalanine | 1.1 |
| Amino Acids | Valine | 1.1 |
| Amino Acids | Glutamic Acid | 1.1 |
| Amino Acids | Leucine | 1.0 |
| Amino Acids | Glutamine | 1.0 |
| Amino Acids | Isoleucine | 1.0 |
| Amino Acids | Threonine | 1.0 |
| Biogenic Amines | Taurine | 2.1 |
| Biogenic Amines | Serotonin | 2.0 |
| Biogenic Amines | alpha-AAA | 1.3 |
| Biogenic Amines | Kynurenine | 1.2 |
| Glycerophospholipids | PC ae C34:2 | 1.6 |
| Glycerophospholipids | PC aa C34:2 | 1.4 |
| Glycerophospholipids | PC ae C36:4 | 1.4 |
| Glycerophospholipids | PC ae C38:5 | 1.3 |
| Glycerophospholipids | PC ae C40:3 | 1.2 |
| Glycerophospholipids | lysoPC a C16:0 | 1.2 |
| Glycerophospholipids | PC ae C42:1 | 1.2 |
| Glycerophospholipids | PC ae C38:3 | 1.2 |
| Glycerophospholipids | PC aa C36:2 | 1.2 |
| Glycerophospholipids | lysoPC a C18:0 | 1.2 |
| Glycerophospholipids | PC aa C40:2 | 1.2 |
| Glycerophospholipids | PC aa C40:3 | 1.2 |
| Glycerophospholipids | PC ae C40:1 | 1.2 |
| Glycerophospholipids | PC ae C40:4 | 1.1 |
| Glycerophospholipids | PC aa C42:1 | 1.1 |
| Glycerophospholipids | PC aa C42:2 | 1.1 |
| Glycerophospholipids | PC ae C42:2 | 1.1 |
| Glycerophospholipids | lysoPC a C20:3 | 1.1 |
| Glycerophospholipids | PC aa C40:1 | 1.1 |
| Glycerophospholipids | PC ae C38:2 | 1.1 |
| Glycerophospholipids | PC aa C24:0 | 1.1 |
| Glycerophospholipids | PC aa C42:4 | 1.1 |
| Glycerophospholipids | PC ae C44:3 | 1.1 |
| Glycerophospholipids | PC aa C42:0 | 1.1 |
| Glycerophospholipids | PC ae C42:3 | 1.0 |
| Glycerophospholipids | PC ae C42:0 | 1.0 |
| Glycerophospholipids | PC ae C38:1 | 1.0 |
| Glycerophospholipids | PC aa C42:6 | 1.0 |
| Glycerophospholipids | PC ae C38:6 | 1.0 |
| Glycerophospholipids | PC ae C30:2 | 1.0 |
| Glycerophospholipids | PC aa C42:5 | 1.0 |
| Glycerophospholipids | PC ae C40:2 | 1.0 |
| Glycerophospholipids | lysoPC a C26:0 | 1.0 |
| Glycerophospholipids | lysoPC a C24:0 | 1.0 |
| Glycerophospholipids | lysoPC a C28:0 | 1.0 |
| Sphingolipids | SM (OH) C14:1 | 1.3 |
| Sphingolipids | SM C20:2 | 1.3 |
| Sphingolipids | SM (OH) C16:1 | 1.2 |
| Sphingolipids | SM C18:1 | 1.2 |
| Sphingolipids | SM C16:1 | 1.1 |
| Sphingolipids | SM (OH) C22:2 | 1.1 |
| Sphingolipids | SM C18:0 | 1.0 |
