# Supplementary material for: Metabolomics Reveals New Mechanisms for Pathogenesis in Barth Syndrome and Introduces Novel Roles for Cardiolipin in Cellular Function
Source: PLoS One. 2016 Mar 25;11(3):e0151802. doi: 10.1371/journal.pone.0151802 (PMC4807847; doi:10.1371/journal.pone.0151802)
Supplement: S3 Table — A positive fold change indicates that the mean concentration of the analyte was higher in BTHS than controls. (DOCX) [file pone.0151802.s003.docx]

Supplementary Table 3. The metabolites and custom ratios quantified by LC-MS using the p180 Biocrates Kit that were either statistically significant (p < 0.05) between BTHS and controls based on the t-test using the Satterthwaite approximation for unequal variances or where the magnitude of the fold change was greater than 2. A positive fold change indicates that the mean concentration of the analyte was higher in BTH than controls.

| Classification | Analyte | p-value | Fold Change |
| --- | --- | --- | --- |
| Acylcarnitines | C2 | 0.003 | 2.1 |
| Acylcarnitines | C14 | 0.011 | 1.2 |
| Acylcarnitines | C3-DC (C4-OH) | 0.030 | 1.3 |
| Acylcarnitines | C5 | 0.041 | 1.3 |
| Acylcarnitines | C4 | 0.043 | 1.4 |
| Acylcarnitines | C12:1 | 0.048 | 1.2 |
| Amino Acids | Proline | 0.001 | 1.5 |
| Amino Acids | Glycine | 0.003 | -1.2 |
| Amino Acids | Arginine | 0.003 | -1.4 |
| Amino Acids | Methionine | 0.007 | 1.9 |
| Amino Acids | Tyrosine | 0.008 | 1.4 |
| Amino Acids | Valine | 0.038 | 1.2 |
| Amino Acids | Citrulline | 0.354 | -2.0 |
| Biogenic Amines | alpha-AAA | 0.007 | 2.1 |
| Biogenic Amines | Kynurenine | 0.044 | 1.2 |
| Biogenic Amines | Serotonin | 0.044 | -8.2 |
| Biogenic Amines | Taurine | 0.072 | -2.8 |
| Biogenic Amines | Carnosine | 0.276 | -2.5 |
| Custom Ratios | Glycine / Serine | <.0001 | -1.3 |
| Custom Ratios | PC aa C28:1 / PC aa C40:2 | <.0001 | 2.9 |
| Custom Ratios | (C2+C3) / C0 | 0.000 | 2.2 |
| Custom Ratios | C2 / C0 | 0.000 | 2.3 |
| Custom Ratios | Total AC / C0 | 0.000 | 1.7 |
| Custom Ratios | PC aa C28:1 / PC aa C38:1 | 0.001 | 2.3 |
| Custom Ratios | lysoPC a C16:0 / lysoPC a C16:1 | 0.002 | -1.3 |
| Custom Ratios | C12 / C8 | 0.002 | 1.2 |
| Custom Ratios | Glycine / Histidine | 0.007 | -1.2 |
| Custom Ratios | C9 / C14 | 0.009 | -1.2 |
| Custom Ratios | Total AC-DC / Total AC | 0.009 | -1.4 |
| Custom Ratios | C4 / C0 | 0.012 | 1.6 |
| Custom Ratios | lysoPC a C20:4 / lysoPC a C20:3 | 0.013 | 1.3 |
| Custom Ratios | C12 / C10 | 0.015 | 1.2 |
| Custom Ratios | Total lysoPC | 0.018 | -1.3 |
| Custom Ratios | Kynurenine / Tryptophan | 0.020 | 1.2 |
| Custom Ratios | Serotonin / Tryptophan | 0.021 | -9.1 |
| Custom Ratios | PC ae C44:5 / PC ae C42:5 | 0.023 | 1.4 |
| Custom Ratios | CPT-I ratio | 0.025 | 1.2 |
| Custom Ratios | Total SM-OH / Total SM-non OH | 0.028 | 1.1 |
| Custom Ratios | Tyrosine / Phenylalanine | 0.029 | 1.2 |
| Custom Ratios | Total lysoPC / Total PC | 0.029 | -1.4 |
| Custom Ratios | Total AC-OH / Total AC | 0.037 | -1.4 |
| Custom Ratios | AAA | 0.039 | 1.2 |
| Custom Ratios | C14:1-OH / C10 | 0.044 | 1.2 |
| Custom Ratios | DOPA/Tyrosine | 0.044 | -1.5 |
| Custom Ratios | Methionine-SO / Methionine | 0.190 | -2.3 |
| Custom Ratios | Glycine / Glutamine | 0.301 | -3.8 |
| Custom Ratios | Glutamic/Glutamine | 0.318 | -4.0 |
| Custom Ratios | Glutaminolysis | 0.325 | -3.4 |
| Custom Ratios | (Leucine+Glutamic)/Glutamine | 0.328 | -3.8 |
| Glycerophospholipids | lysoPC a C20:3 | 0.003 | -1.6 |
| Glycerophospholipids | PC aa C32:1 | 0.003 | 1.6 |
| Glycerophospholipids | PC ae C32:1 | 0.005 | 1.4 |
| Glycerophospholipids | PC ae C34:1 | 0.006 | 1.4 |
| Glycerophospholipids | PC aa C40:4 | 0.006 | -1.3 |
| Glycerophospholipids | lysoPC a C18:0 | 0.007 | -1.4 |
| Glycerophospholipids | PC ae C34:0 | 0.013 | 1.4 |
| Glycerophospholipids | lysoPC a C16:0 | 0.013 | -1.3 |
| Glycerophospholipids | PC ae C38:5 | 0.013 | 1.4 |
| Glycerophospholipids | PC aa C28:1 | 0.017 | 1.4 |
| Glycerophospholipids | PC ae C34:2 | 0.017 | 1.3 |
| Glycerophospholipids | PC aa C30:0 | 0.019 | 1.4 |
| Glycerophospholipids | PC ae C38:0 | 0.021 | -1.5 |
| Glycerophospholipids | PC aa C32:0 | 0.023 | 1.2 |
| Glycerophospholipids | PC ae C40:3 | 0.029 | -1.8 |
| Glycerophospholipids | PC ae C40:1 | 0.038 | -1.5 |
| Glycerophospholipids | PC ae C36:4 | 0.040 | 1.3 |
| Glycerophospholipids | PC aa C42:6 | 0.041 | -1.3 |
| Glycerophospholipids | PC ae C40:4 | 0.042 | -1.4 |
| Glycerophospholipids | PC aa C36:6 | 0.042 | -1.4 |
| Glycerophospholipids | lysoPC a C24:0 | 0.043 | -1.4 |
| Glycerophospholipids | PC aa C42:4 | 0.044 | -1.7 |
| Glycerophospholipids | PC aa C24:0 | 0.047 | -1.4 |
| Glycerophospholipids | PC aa C42:5 | 0.048 | -1.3 |
| Glycerophospholipids | PC aa C40:2 | 0.081 | -2.0 |
| Sphingolipids | SM C20:2 | 0.003 | 1.4 |
| Sphingolipids | SM (OH) C14:1 | 0.006 | 1.5 |
| Sphingolipids | SM (OH) C22:2 | 0.029 | 1.3 |
| Sphingolipids | SM (OH) C16:1 | 0.044 | 1.3 |
