# Supplementary figures and images for: Metabolomics Reveals New Mechanisms for Pathogenesis in Barth Syndrome and Introduces Novel Roles for Cardiolipin in Cellular Function
Source: PLoS One. 2016 Mar 25;11(3):e0151802. doi: 10.1371/journal.pone.0151802 (PMC4807847; doi:10.1371/journal.pone.0151802)

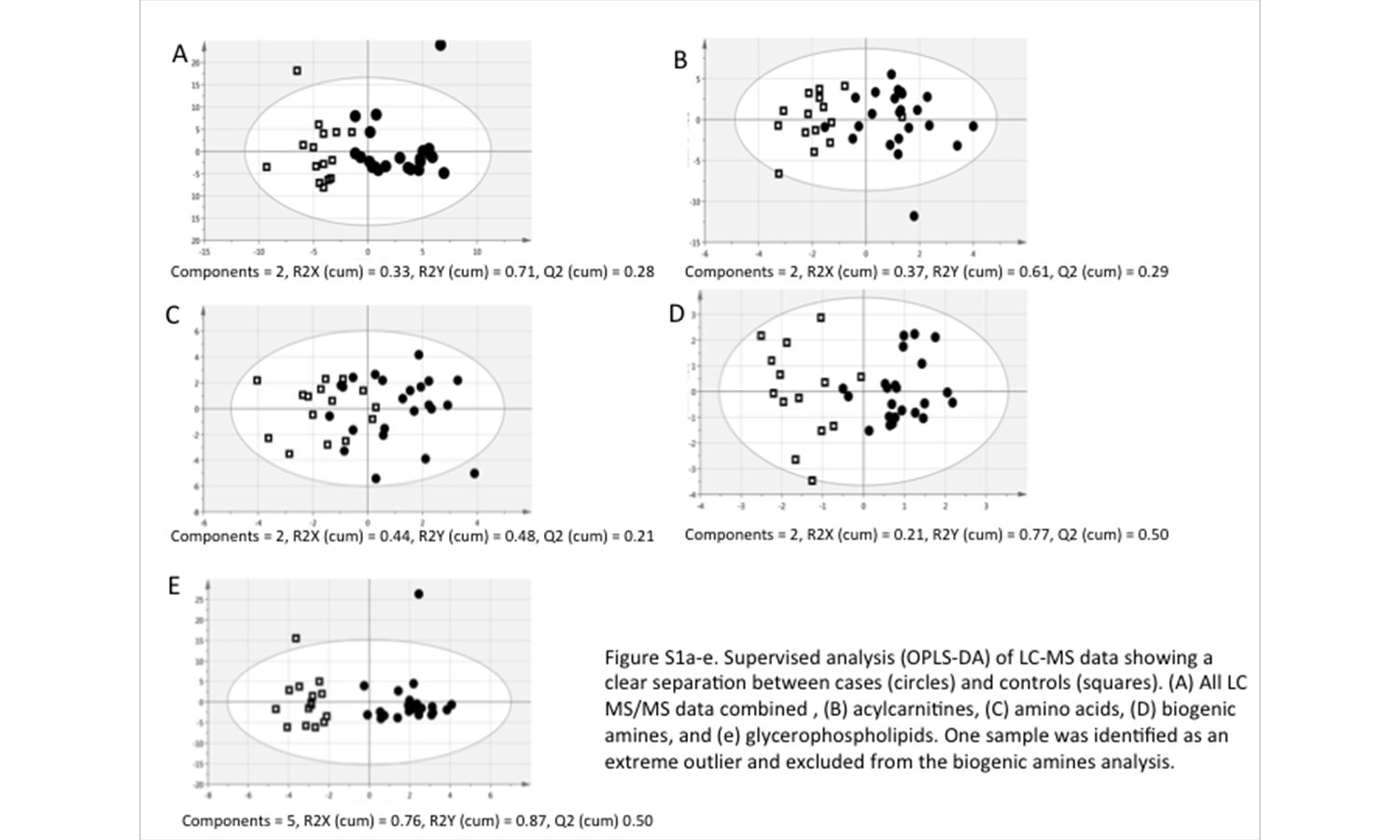

Supplement: S1 Fig — (A) All LC MS/MS data combined, (B) acylcarnitines, (C) amino acids, (D) biogenic amines, and (e) glycerophospholipids. One sample was identified as an extreme outlier and excluded from the biogenic amines analysis (TIFF) [file pone.0151802.s004.tiff]

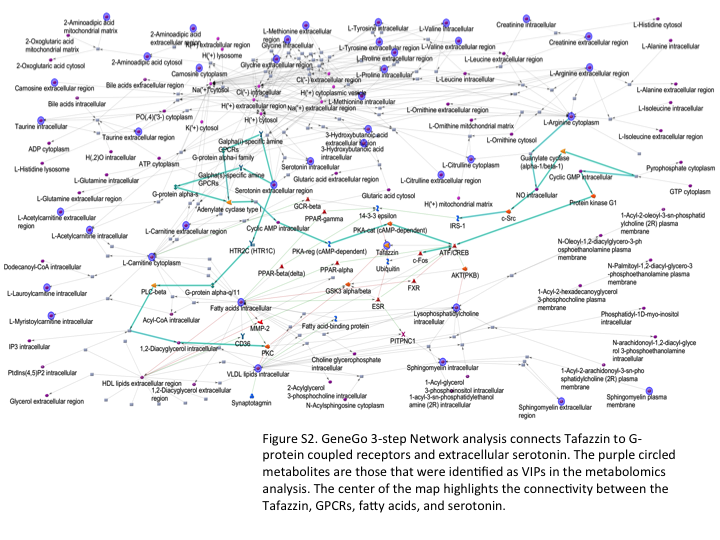

Supplement: S2 Fig — The purple circled metabolites are those that were identified as VIPs in the metabolomics analysis. The center of the map highlights the connectivity between the Tafazzin, GPCRs, fatty acids, and serotonin. (TIFF) [file pone.0151802.s005.tiff]
